# Widely tunable GaAs bandgap via strain engineering in core/shell nanowires with large lattice mismatch

**DOI:** 10.1038/s41467-019-10654-7

**Published:** 2019-06-26

**Authors:** Leila Balaghi, Genziana Bussone, Raphael Grifone, René Hübner, Jörg Grenzer, Mahdi Ghorbani-Asl, Arkady V. Krasheninnikov, Harald Schneider, Manfred Helm, Emmanouil Dimakis

**Affiliations:** 1 Institute of Ion Beam Physics and Materials Research, Helmholtz-Zentrum Dresden-Rossendorf, 01328 Dresden, Germany; 2 Center for Advancing Electronics Dresden (cfaed), Technische Universität Dresden, 01062 Dresden, Germany; 3 PETRA III, Deutsches Elektronen-Synchrotron (DESY), 22607 Hamburg, Germany

**Keywords:** Nanophotonics and plasmonics, Nanowires

## Abstract

The realisation of photonic devices for different energy ranges demands materials with different bandgaps, sometimes even within the same device. The optimal solution in terms of integration, device performance and device economics would be a simple material system with widely tunable bandgap and compatible with the mainstream silicon technology. Here, we show that gallium arsenide nanowires grown epitaxially on silicon substrates exhibit a sizeable reduction of their bandgap by up to 40% when overgrown with lattice-mismatched indium gallium arsenide or indium aluminium arsenide shells. Specifically, we demonstrate that the gallium arsenide core sustains unusually large tensile strain with hydrostatic character and its magnitude can be engineered via the composition and the thickness of the shell. The resulted bandgap reduction renders gallium arsenide nanowires suitable for photonic devices across the near-infrared range, including telecom photonics at 1.3 and potentially 1.55 μm, with the additional possibility of monolithic integration in silicon-CMOS chips.

## Abstract (web summary)

Designing core/shell nanowires with desired optoelectronic properties of III-V semiconductor alloys remains a challenge. Here, the authors report an engineering strategy to surmount strain-induced difficulties in the growth achieving highly strained cores with a sizeable change in their band gap.

## Introduction

III-V compound semiconductors have stimulated many breakthroughs in physics and technology owing to their direct bandgap and high electron mobility^1^. It has also been very important that these fundamental properties can be tailored, depending on the targeted device application or operation energy-range, by using (qua-) ternary alloys with selected chemical compositions. In_*x*_Ga_1−*x*_As is a representative example for applications in the near-infrared range, where the In-content *x* can be chosen to provide appropriate bandgaps for multi-junction photovoltaics, light emitting diodes and photodiodes, or telecom photonics^2,3^. Nevertheless, not all compositions and corresponding bandgaps between the two endpoint binaries of a ternary alloy (e.g. GaAs and InAs for In_*x*_Ga_1−*x*_As) are feasible because of the unavailability of lattice-matched substrates as well as the spinodal decomposition^4^. Furthermore, the alloy disorder is another factor that affects the performance of ternary alloys^5,6^.

More recently, III–V semiconductors in the form of free-standing nanowires have shown new potentials for a wide range of future applications in nanotechnology, e.g., photovoltaic cells with enhanced light absorption^7^, lasers with sub-wavelength size^8^, tunnel field-effect transistors as energy-efficient electronic switches^9^, and entangled photon-pair sources for quantum information technology^10^. Owing to their small footprint, nanowires can also be grown epitaxially without dislocations on lattice-mismatched substrates, enabling the monolithic integration of dissimilar materials with complementary properties, such as III–V semiconductors and Si^11–14^ or graphene^15,16^. A distinct feature of the nanowire geometry is the possibility to create core/shell heterostructures of highly lattice-mismatched materials well beyond the limits for coherent growth in equivalent thin-film heterostructures^17^. The lattice mismatch can be accommodated via elastic deformation of not only the shell, but also the core, depending on the relative thicknesses and chemical compositions^18^. This increases the capabilities for engineering the strain and, thus, the electronic structure and properties of the heterostructure^19–23^. Unlike quantum-dot heterostructures, where elastic accommodation of large misfit stresses is also possible^24^, the hetero-interface in nanowires can be several micrometres long, allowing for practical use in a wide variety of device concepts, e.g., in photovoltaics, lasers, thermoelectrics and electronics^25^.

Strain-induced changes in the bandgap of the core in core/shell nanowires have been reported for GaAs/GaP^26^, GaAs/Al_x_Ga_1−x_As^27^, InAs/InAs_x_P_1−x_^22^, and GaN/Al_x_Ga_1−x_N^20^. In all cases, the core was compressively strained and its bandgap increased, in the most extreme case by 260 meV^26^. Tensile strain and up to 150 meV smaller bandgap in the core have been reported only for GaAs/Ga_x_In_1−x_P nanowires^28^. Nevertheless, extending the same concept to higher strain values is not straightforward owing to limiting factors like plastic relaxation and/or morphological instabilities^29–32^. Alternatively, quantum confinement in thin nanowires^33^ or post-growth external stress^34,35^, which is less practical though for device applications, have also been suggested for tuning the GaAs bandgap.

Here, we exploit the unique opportunities for strain engineering in nanowires to achieve wide tuning of the bandgap in GaAs, a traditional III–V binary alloy. Specifically, we investigate the strain in highly-mismatched GaAs/In_*x*_Ga_1−*x*_As and GaAs/In_*x*_Al_1−*x*_As core/shell nanowires, and its effects on the electronic properties of the GaAs core. The nanowires are grown epitaxially on Si substrates. Our work shows how to surmount strain-induced difficulties in the growth, how the misfit strain is distributed between the core and the shell depending on the design of the heterostructure and, most important, how to obtain highly strained cores with a sizeable change in their bandgap. After all, we demonstrate the possibility to reduce the bandgap of GaAs by up to 40% (≈600 meV) in a continuous manner (all intermediate values are possible), which renders GaAs nanowires a versatile material system for various photonic devices in the near-infrared range, including the 1.3 μm and potentially the 1.55 μm telecom windows, monolithically integrated on the same Si chip.

## Results

### Growth of strained core/shell nanowires

**Fig. 1:**
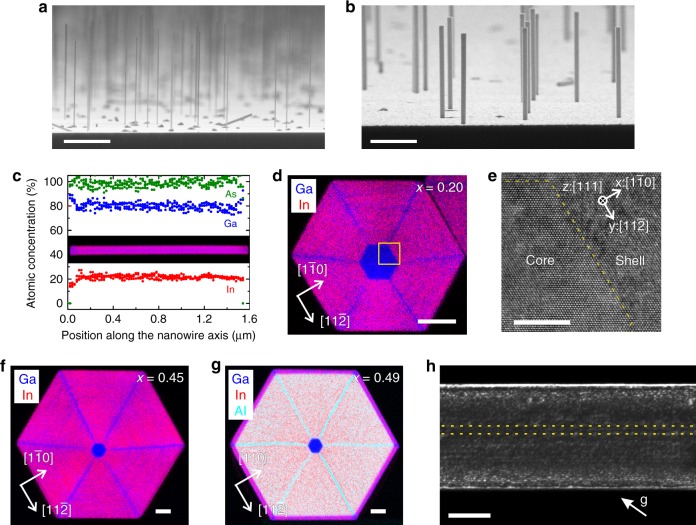
Morphological, compositional and structural analysis of GaAs/In_*x*_Ga_1−*x*_As and GaAs/In_*x*_Al_1−*x*_As core/shell nanowires grown on Si(111) substrates. **a** Side-view scanning electron microscopy (SEM) image of as-grown bare GaAs core nanowires and (**b**) GaAs/In_*x*_Ga_1−*x*_As core/shell nanowires (*x* = 0.20, shell thickness *L*_S_ = 40 nm). **c** Energy-dispersive X-ray spectroscopy (EDXS) compositional analysis of the shell along the axis of one nanowire from the sample shown in (**b**). The zero position corresponds to the tip of the nanowire. The inset depicts the corresponding compositional map. **d** EDXS compositional map perpendicular to the axis of one nanowire from the sample shown in (**b**). **e** High-resolution transmission electron microscopy (TEM) micrograph of the core/shell interface region shown in (**d**) with a yellow square. The dotted line indicates the core/shell interface. The $\left[ {1\bar 10} \right]$, $\left[ {11\bar 2} \right]$ and $\left[ {111} \right]$ crystallographic directions are indicated as *x*-, *y*- and *z*- axes, respectively. **f** EDXS compositional map perpendicular to the axis of a GaAs/In_*x*_Ga_1−*x*_As core/shell nanowire with *x* = 0.45 and *L*_S_ = 80 nm. **g** EDXS compositional map perpendicular to the axis of a GaAs/In_*x*_Al_1−*x*_As core/shell nanowire with *x* = 0.49 and *L*_S_ = 80 nm. **h** (220) weak-beam dark-field TEM image of a GaAs/In_*x*_Al_1−*x*_As nanowire like in (**g**) that shows no misfit dislocations in the region of the core (between the yellow dotted lines). The scale bars correspond to 1 μm in (**a**, **b**), 30 nm in (**d**, **f**, **g**), 5 nm in (**e**), and 100 nm in (**h**)

Vertical GaAs/In_*x*_Ga_1−*x*_As and GaAs/In_*x*_Al_1−*x*_As core/shell nanowires were grown on Si(111) substrates by molecular beam epitaxy (MBE). First, GaAs core nanowires with a diameter of 20–25 nm and a length of 2 μm were grown in self-catalysed mode^36^ and then In_*x*_Ga_1−*x*_As or In_*x*_Al_1−*x*_As shells were grown around the core nanowires (see Methods). The shell thickness (*L*_S_) and composition (*x*) were varied independently according to the needs of our study. Figure 1a, b depict side-view scanning electron microscopy (SEM) images of bare GaAs core nanowires (without shell) and GaAs/In_*x*_Ga_1−*x*_As core/shell nanowires (*x* = 0.20, *L*_S_ = 40 nm), respectively. The growth conditions for the shell were tuned to obtain a homogeneous thickness and composition around the core nanowires. Specifically, the growth of the shell was performed at a considerably low substrate temperature (370 °C) with a continuous substrate-rotation of 20 rpm and relatively high growth rates (≈ 0.6 Å/s). Apart from the nanowires, a continuous planar layer with polycrystalline structure and similar composition to that of the nanowire shells also grew on the substrate.

The structure and composition of the nanowires were evaluated with transmission electron microscopy (TEM). The nanowires grew along the $[\bar 1\bar 1\bar 1]$ crystallographic direction and have six $\left\{ {1\bar 10} \right\}$ sidewalls. Figure 1c, d show element maps along and perpendicular to the nanowire axis as measured with energy-dispersive X-ray spectroscopy (EDXS; see Methods). The incorporation of In into the shell was found very homogeneous except for the nanowire corners (Fig. 1d), where the incorporation was reduced, giving rise to six $< 11\bar 2 >$ lines of lower *x* (the same occurs in In_*x*_Al_1−*x*_As shells). Similar phenomena have been observed by others in various material systems and have been attributed to self-ordering effects that occur during heteroepitaxy on nonplanar substrates^37^. The shell adopted the crystal structure of the core (see Supplementary Fig. 1 and Supplementary Note 1), i.e., both the core and the shell grew in the zinc blende structure and only the two ends of the nanowires contain a high number of rotational twins around the $\left[ {111} \right]$ nanowire axis and stacking faults that were formed in the beginning and the end of the GaAs growth (owing to transient changes of the droplet contact angle). Small wurtzite segments (i.e. continuous formation of twins) could be found by high-resolution TEM only occasionally and only at the two ends of the nanowires, but their volume was negligible compared to the total volume of the nanowires. Finally, the coherent growth along the $\langle11\bar 2\rangle$ crystallographic directions of the core/shell interface was evidenced by the absence of misfit dislocations as shown in Fig. 1e (high-resolution TEM image of the region shown with the yellow square in Fig. 1d).

We obtained similar results also for In_*x*_Ga_1−*x*_As shells with higher *x* and larger *L*_S_ or for In_*x*_Al_1−*x*_As shells. For example, cross-sectional EDXS element maps for In_0.45_Ga_0.55_As and In_0.49_Al_0.51_As shells (*L*_S_ = 80 nm) are shown in Fig. 1f, g, respectively. The absence of misfit dislocations at the core/shell interface across the whole nanowire length was confirmed with TEM weak-beam dark-field measurements using the 220 reflection and the so-called (g, 3 g) condition. The example of a GaAs/In_0.49_Al_0.51_As nanowire, of the same type like the one in Fig. 1g, is shown in Fig. 1h. Finally, we found that the highest possible *x* for coherent growth of GaAs/In_*x*_Ga_1−*x*_As nanowires with *L*_S_ = 80 nm resides between 0.55 (no misfit dislocations were observed) and 0.70 (misfit dislocations were observed).

It is worth to mention that we identified a tendency for preferential growth of the shells with high *x* on one side of the core, similar to Day et al.^38^ for highly strained Si/Ge core/shell nanowires. This effect imposed anisotropic misfit stress to the core and, thus, the nanowires bent towards the thinner shell side^21,30,38,39^. However, it was possible to minimise this tendency by performing the shell growth at sufficiently low temperatures with high growth rates, which imposed strong kinetic limitations. In contrast, the nanowires were bent permanently at higher growth temperatures and lower growth rates, even for *x* as low as 0.20.

### Analysis of strain in core/shell nanowires

**Fig. 2:**
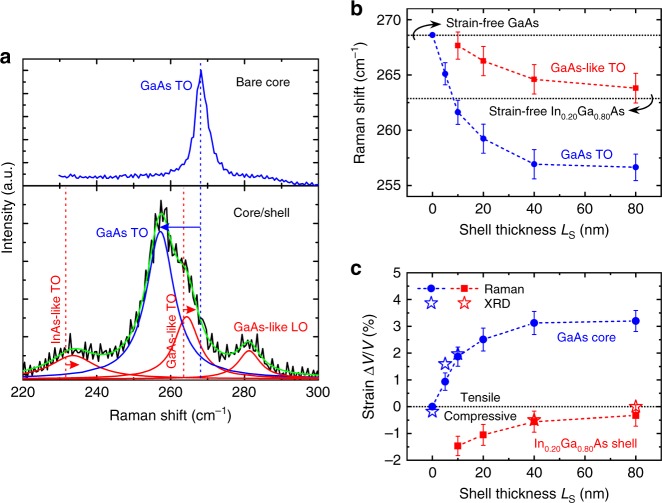
Strain analysis of GaAs/In_0.20_Ga_0.80_As core/shell nanowires as a function of shell thickness *L*_S_. **a** Raman scattering spectra at 300 K from a single core nanowire without shell (“bare core”) and a single core/shell nanowire with *L*_S_ = 40 nm (“core/shell”). Phonon peaks in blue are attributed to the core, whereas the ones in red belong to the shell. The cumulative fit curve is shown in green and the strain-free values of phonons with vertical dashed lines. The arrows indicate the strain-induced shift of the various transverse optical (TO) phonons. **b** Raman shift of the GaAs (blue data points) and the GaAs-like (red data points) TO phonons as a function of *L*_S_. The dashed lines are guides to the eye, whereas the horizontal dotted lines show the expected Raman shift of GaAs and GaAs-like TO phonons in strain-free GaAs and strain-free In_0.20_Ga_0.80_As, respectively. The error bars represent the standard deviation and the instrument error. **c** The hydrostatic strain in the core (blue data points) and the shell (red data points) as a function of *L*_S_. The dashed lines are guides to the eye. The error bars originate from the error bars in Raman shift. The star symbols correspond to X-ray diffraction (XRD) results

The strain in core/shell nanowires was measured by micro-Raman scattering spectroscopy at 300 K (*λ* = 532 nm, beam spot size = 800 nm). The measurements were performed in back-scattering configuration with normal incidence excitation on single nanowires (see Methods), which had been transferred previously on an Au-coated Si wafer. The removal of nanowires from their original substrate did not affect their strain state because of the small nanowire/substrate interface area (see Supplementary Fig. 2d and Supplementary Note 2). An example of a GaAs/In_*x*_Ga_1−*x*_As core/shell nanowire with *x* = 0.20 and *L*_S_ = 40 nm is shown in Fig. 2a in comparison with a bare GaAs nanowire (without shell). The spectrum of the bare GaAs nanowire is dominated by scattering from transverse optical (TO) phonons, with the peak position at 268.6 cm^−1^ in good agreement with reported values for strain-free bulk GaAs in zinc blende phase^40,41^ (longitudinal optical (LO) phonon transitions are forbidden in the particular measurement geometry, but a weak signal is still present). In contrast, the spectrum of the core/shell nanowire shows a more complex structure. Using Lorentzian curves for the fitting of the line shape, we identified three scattering contributions, i.e. scattering in the core from GaAs TO phonons and scattering in the shell from GaAs-like and InAs-like TO phonons (see Methods). Measuring the relative peak shift (Δ*ω*/*ω*) with respect to the strain-free position for GaAs and GaAs-like TO phonons (268.6 cm^−1^ and 268.6–30·*x*  cm^−1^, respectively^42,43^), we deduced the amount of hydrostatic strain Δ*V*/*V* in the core and in the shell, respectively, using the following equation:

1 $${\mathrm{\Delta }}V/V = 1/\gamma _{{\mathrm{TO}}} \cdot {\mathrm{\Delta }}\omega /\omega ,$$

where *γ*_TO_ = 1.39 is the hydrostatic deformation potential (or Grüneisen parameter) of GaAs TO phonons^40^. Here, the shift of GaAs TO phonons has been attributed exclusively to strain, neglecting any other potential contributions (e.g., phonon confinement or zone-folding)^44,45^. This means that our Δ*V*/*V* values for the GaAs core correspond to the highest possible strain. Given the large size of the laser beam compared to the nanowire length, the potential existence of alloy disorder in the shell (below the resolution of our EDXS analysis) or any other type of local structural disorder is expected to affect the width of the phonon lines rather than their peak positions.

Figure 2b, c summarise the measured Raman shifts and the corresponding strain, respectively, in GaAs/In_*x*_Ga_1−*x*_As core/shell nanowires as a function of *L*_S_ for *x* = 0.20 (see all Raman spectra in Supplementary Fig. 3a). For the smallest *L*_S_, both the shell and the core are strained, i.e., the shell is compressively strained, whereas the core is tensile-strained. With increasing *L*_S_, though, the shell becomes less strained and the core more strained. In other words, the compressive misfit strain that exists in thin shells is elastically relaxed with increasing *L*_S_ by stretching the core (later we will show that this is not the only mechanism of strain relaxation in the shell). Eventually, for *L*_S_ ≥ 40 nm the shell becomes almost strain-free, whereas the strain in the core saturates at 3.2%. These results already show that thin enough nanowires can be used as flexible substrates for overgrowth with lattice-mismatched shells, going far beyond what is possible in equivalent thin-film heterostructures^46^.

**Fig. 3:**
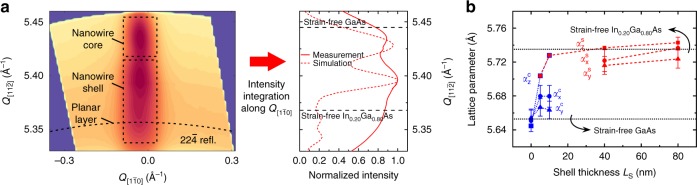
Measurements of the lattice parameters in GaAs/In_0.20_Ga_0.80_As core/shell nanowires as a function of shell thickness *L*_S_ by high-resolution X-ray diffraction (XRD). The measurements were performed on ensembles of as-grown nanowires. **a** Left: An example of a 2D reciprocal space map of the $22\bar 4$ reflection for nanowires with *L*_S_ = 10 nm. The contributions from the core and the shell are indicated with dashed rectangles. The weak contribution from the planar polycrystalline layer is indicated with a curved dashed line. Right: the corresponding 1D plot after integration of the 2D map intensity along $Q_{[1\bar 10]}$. The identification of the core and shell contributions was based on the comparison of the measurement (continuous curve) with the theoretical simulation (dashed curve). The multiple peaks from the shell in the range of 5.33–5.40 Å^−1^ indicate the complex strain distribution in thin shells. The horizontal dashed lines indicate the strain-free values of the $22\bar 4$ reflection for GaAs and In_0.20_Ga_0.80_As. **b** XRD-measured average lattice parameters of the core (blue data points) and the shell (red data points) as a function of *L*_S_. $\alpha _{\mathrm{z}}$ is the lattice parameter parallel to the nanowire axis, extracted from the $\bar 1\bar 1\bar 1$ reflection. $\alpha _{\mathrm{x}}$ and $\alpha _{\mathrm{y}}$ are two orthogonal lattice parameters perpendicular to the nanowire axis, extracted from the $20\bar 2$ and the $22\bar 4$ reflections, respectively. The dashed lines are guides to the eye, whereas the horizontal dotted lines show the strain-free lattice parameters for GaAs and In_0.20_Ga_0.80_As. The error bars originate from the fitting error of the corresponding 1D XRD spectra

The strain state of the GaAs core and the In_0.20_Ga_0.80_As shell was verified using high-resolution X-ray diffraction (XRD) at synchrotron light sources. The lattice parameters of the core and the shell were measured along the three orthogonal crystallographic directions x, y, z defined in Fig. 1e (*z*-axis is parallel to the nanowire axis, whereas *x*- and *y*- axes are perpendicular to it). For this purpose, three-dimensional reciprocal space maps were recorded for nanowire ensembles around the $20\bar 2$, $22\bar 4$ and $\bar 1\bar 1\bar 1$ Bragg reflections, respectively (see Methods). As an example, the reciprocal space map around the $22\bar 4$ reflection, projected on the $(Q_{[11\bar 2]},\,Q_{[1\bar 10]})$ plane, for nanowires with *L*_S_ = 10 nm is depicted in Fig. 3a. The contributions from the core and the shell are indicated with dashed rectangles. The corresponding 1D plot along $Q_{[11\bar 2]}$, after integration of the intensity along $Q_{[1\bar 10]}$, is also shown in Fig. 3a. The comparison of the 1D plot (continuous curve) with simulations (dashed curve) based on elasticity theory (see Methods) shows a reasonable agreement. Measurements and simulations were also performed on nanowires with *L*_S_ = 0, 5, 40, and 80 nm (see Supplementary Fig. 5). The diffraction signal from the core was strong enough and, thus, could be unambiguously identified only for *L*_S_ *=* 0, 5, and 10 nm. On the other hand, the complexity of the radial strain profile in thin shells (see Supplementary Fig. 6) allowed for extracting single lattice parameters for the shell along x or y direction only for *L*_S_ = 40 and 80 nm. The extracted average lattice parameters of the core ($\alpha _{\mathrm{x}}^{\mathrm{c}}$, $\alpha _{\mathrm{y}}^{\mathrm{c}}$, $\alpha _{\mathrm{z}}^{\mathrm{c}}$) and the shell ($\alpha _{\mathrm{x}}^{\mathrm{s}}$, $\alpha _{\mathrm{y}}^{\mathrm{s}}$, $\alpha _{\mathrm{z}}^{\mathrm{s}}$) are plotted in Fig. 3b as a function of *L*_S_.

The fact that all three lattice parameters of the core increased with *L*_S_ is a manifestation of the hydrostatic character of strain in the core. $\alpha _{\mathrm{z}}^{\mathrm{c}}$, which was found equal to $\alpha _{\mathrm{z}}^{\mathrm{s}}$, increased gradually with *L*_S_ from the value of strain-free GaAs to that of strain-free In_0.20_Ga_0.80_As. This means that for thick enough shells, the misfit along the nanowire axis was elastically accommodated exclusively by stretching the core. The situation is not the same in the x–y plane. $\alpha _{\mathrm{x}}^{\mathrm{c}}$ and $\alpha _{\mathrm{y}}^{\mathrm{c}}$ also increased with *L*_S_, but they showed a tendency to saturate well below the value of strain-free In_0.20_Ga_0.80_As. Nevertheless, $\alpha _{\mathrm{x}}^{\mathrm{s}}$ and $\alpha _{\mathrm{y}}^{\mathrm{s}}$ reached almost strain-free values, which suggests that the misfit perpendicular to the nanowire axis was only partly accommodated by stretching the core. We speculate that the reason for that is the continuously increasing width of the nanowire sidewalls during shell growth, which also enables the elastic accommodation of misfit.

The strain components along ($\varepsilon _{{\mathrm{zz}}}$) and perpendicular ($\varepsilon _{{\mathrm{xx}}}$, $\varepsilon _{{\mathrm{yy}}}$) to the nanowire axis were calculated for the GaAs core as $\varepsilon _{{n}} = \left( {\alpha _{{n}}^{\mathrm{c}} - \alpha _0} \right)/\alpha _0$ (where ${{n}} = {\mathrm{xx}},\,{\mathrm{yy}},\,{\mathrm{zz}}$, and *α*_0_ = 5.6533 Å is the lattice parameter of strain-free GaAs), whereas the corresponding hydrostatic strain was calculated as ${\mathrm{\Delta }}V/V = \varepsilon _{{\mathrm{xx}}} + \varepsilon _{{\mathrm{yy}}} + \varepsilon _{{\mathrm{zz}}}$. As shown in Fig. 2c, the results for Δ*V*/*V* (star symbols) are in good agreement with the strain measured by Raman scattering. Thus, it is two independent experimental techniques that verify the unusually large strain in the GaAs core.

**Fig. 4:**
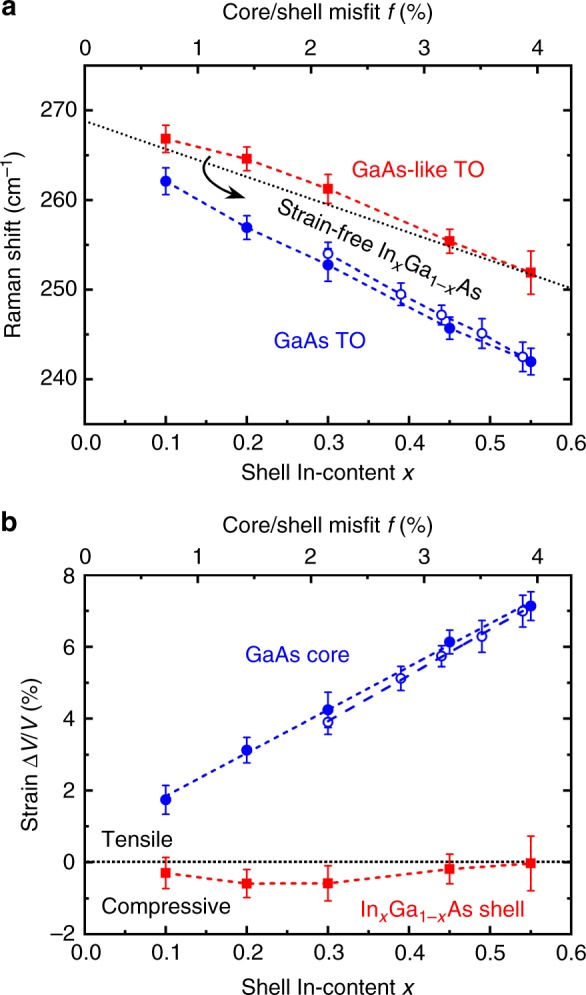
Strain analysis of GaAs/In_*x*_Ga_1−*x*_As (shell thickness *L*_S_ = 40–80 nm; closed symbols) and GaAs/In_*x*_Al_1−*x*_As (*L*_S_ = 80 nm; open symbols) core/shell nanowires as a function of In-content *x* in the shell (bottom *x*-axis) and the corresponding core/shell misfit *f* (top *x*-axis). **a** Raman shift of the GaAs (blue data points) and the GaAs-like (red data points) transverse optical (TO) phonons as a function of *x* and *f*. The dashed lines are guides to the eye, whereas the dotted line shows the expected Raman shift of GaAs-like TO phonons in strain-free In_*x*_Ga_1−*x*_As. The error bars represent the standard deviation and the instrument error. GaAs-like TO phonons do not exist in In_*x*_Al_1−*x*_As shells. **b** The hydrostatic strain in the core (blue data points) and the shell (red data points) as a function of *x* and *f*. The blue dashed lines are linear fits, whereas the red one is a guide to the eye. The error bars originate from the error bars in Raman shift

The amount of tensile strain in the GaAs core depends also on the core/shell misfit *f* (relative difference in lattice constants) or, in other words, the shell composition *x*. Figure 4a summarises the Raman shift and Fig. 4b the corresponding strain inGaAs/In_*x*_Ga_1−*x*_As core/shell nanowires with different values of *x*, from 0.10 to 0.55 (the nominal values of *x* were confirmed for a selected number of samples by EDXS analysis in the TEM), and *L*_S_ = 40–80 nm (see all Raman spectra in Supplementary Fig. 3b). The tensile strain in the core was found to increase linearly with *x* (i.e. Δ*V*/*V* = 1.7 *f*), whereas the shell remained approximately strain-free. The tensile strain in the core was also measured for GaAs/In_*x*_Al_1−*x*_As nanowires with different *x* and *L*_S_ = 80 nm (see all Raman spectra in Supplementary Fig. 4). The results (open symbols in Fig. 4; Δ*V*/*V* = 1.8 *f*) are similar to those for GaAs/In_*x*_Ga_1−*x*_As nanowires because of the similar lattice parameters of In_*x*_Ga_1−*x*_As and In_*x*_Al_1−*x*_As for the same *x*. Assuming $\varepsilon _{{\mathrm{zz}}} \approx f$, we estimate that the strain in the core is 2.5 times larger along the nanowire axis than perpendicular to it (i.e. $\varepsilon _{{\mathrm{zz}}} = 2.5\,\varepsilon _{{\mathrm{xx}}} = 2.5\,\varepsilon _{{\mathrm{yy}}}$).

For the highest misfit in this work, i.e. *f* = 4% for In_0.55_Ga_0.45_As or In_0.54_Al_0.46_As shells, the tensile hydrostatic strain in the core reached the remarkably large value of 7%. The linear increase of strain in the core with *x* suggests that no apparent plastic relaxation occurred, in accordance with our TEM analysis. These results are in agreement with theoretical predictions of growth coherency in core/shell nanowires with *f* up to 4% and core radii of about 10 nm or less^47^. We point out that it would have been impossible to grow such highly lattice-mismatched heterostructures in conventional thin-film geometry without forming dislocations.

### Effect of strain on the electronic properties of the GaAs core

**Fig. 5:**
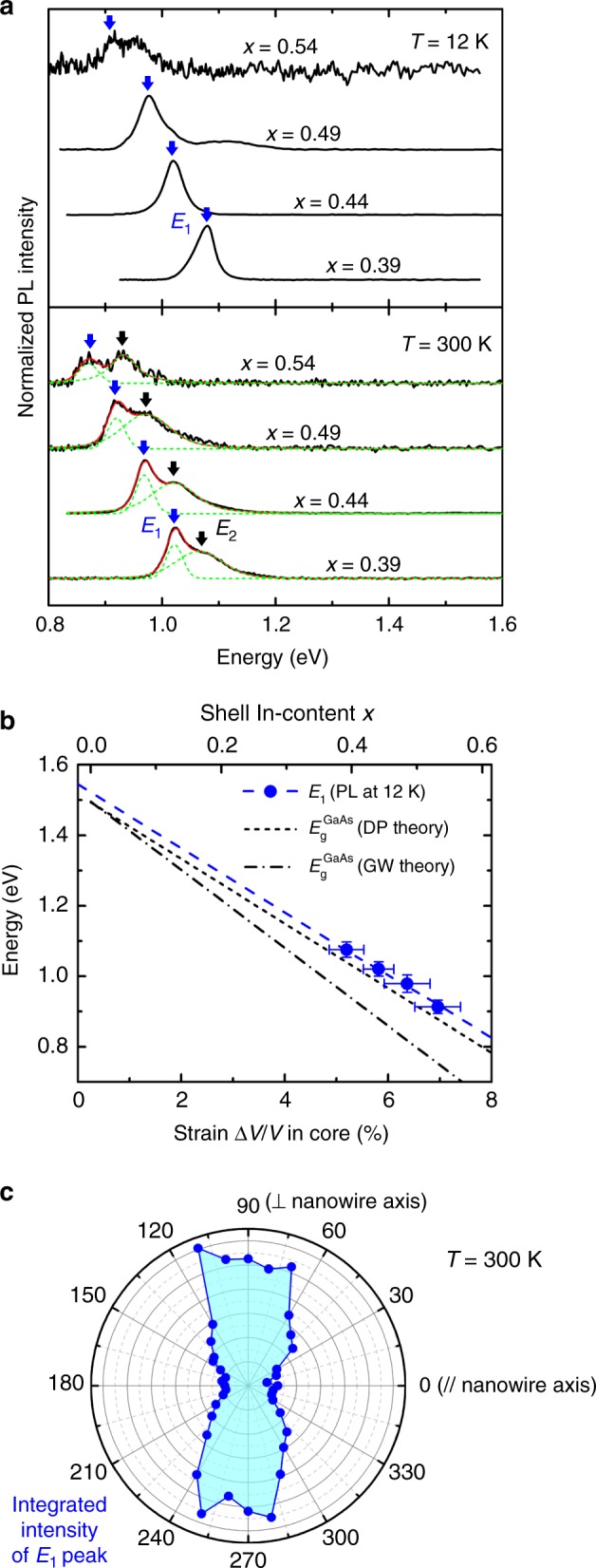
Effect of strain on the bandgap energy of the core in GaAs/In_*x*_Al_1−*x*_As core/shell nanowires with shell thickness *L*_S_ = 80 nm. **a** Photoluminescence (PL) spectra as measured on ensembles of nanowires with different *x* at 12 and 300 K. The spectra have been shifted vertically for the sake of clarity. Fit curves (Voigt) are shown in green (contributing peaks) and red (cumulative curves). Primary (*E*_1_) and secondary (*E*_2_) emissions are indicated with blue and black arrows, respectively. **b** *E*_1_ peak energy at 12 K (blue data points) as a function of the hydrostatic strain in the core (bottom *x*-axis) and the corresponding In-content *x* in the shell (top *x*-axis). The energy error bars correspond to the full-width at half-maximum of the PL fit curves, whereas the strain error bars originate from the error bars in Raman scattering measurements. The blue dashed line is a linear fit of the PL data. The black lines correspond to the bandgap energy of bulk GaAs as a function of strain, calculated with deformation potential theory (DP; dashed line) or from first-principles (GW; dash-dotted line). **c** Integrated intensity of the *E*_1_ peak in polarisation-resolved PL at 300 K. The polarisation of the excitation light was parallel to the nanowire axis (0° and 180°)

The effect of strain on the bandgap of the GaAs core was studied by means of photoluminescence (PL) spectroscopy. The existence of tensile strain with hydrostatic character in the core is expected to reduce the bandgap. In fact, the bandgaps of the tensile-strained GaAs core and the strain-free In_*x*_Ga_1−*x*_As shell are expected to be similar^1,48^, which makes their distinction in optical spectra difficult. To avoid any ambiguities, we used GaAs/In_*x*_Al_1−*x*_As core/shell nanowires, where the larger bandgap of strain-free In_*x*_Al_1−*x*_As (larger than 1.38 eV at 12 K for *x* ≤ 0.54) cannot be confused with that of the tensile-strained GaAs. PL measurements were performed at 12 and 300 K (laser excitation at 532 nm) on ensembles of GaAs/In_*x*_Al_1−*x*_As nanowires, which had been transferred previously on amorphized Ge wafers (to quench the photoluminescence from crystalline Ge). The spectra for different values of *x* (*L*_S_ = 80 nm) are plotted in Fig. 5a. Emission was obtained only in the 0.8–1.2 eV range, which is suggestive of radiative recombination of electron-hole pairs only inside the tensile-strained GaAs core. The emission shifts to lower energies with increasing *x*, in agreement with the expected effect of increasing tensile strain in the core.

The energy of the emission peak (*E*_1_) at 12 K is plotted in Fig. 5b as a function of the hydrostatic strain (Δ*V*/*V*) in the core and the corresponding In-content *x* in the shell. The linear dependence was fitted (blue dashed line) with an equation of the form:

2 $$E_1 = E^0 + a \cdot \frac{{{\mathrm{\Delta }}V}}{V},$$

where *E*^0^ is the strain-free value of *E*_1_ and *a* is the hydrostatic deformation potential of *E*_1_. The fitting parameters (*E*^0^ = 1.55 ± 0.03 eV and *a* = −9.0 ± 0.5 eV) are in good agreement with the strain-free bandgap energy (1.52 eV) and the hydrostatic deformation potential (−8.5 eV) of bulk GaAs^1,49,50^. Thus, *E*_1_ can be attributed to band-edge transitions in the tensile-strained GaAs core.

Our results are compared in Fig. 5b with the bandgap of strained GaAs calculated either from first-principles (a combination of density-functional theory, DFT, with GW approximation; black dash-dotted line) or with the band-edge deformation potential (DP) theory (black short-dashed line)^49,51^. The theoretical bandgap here is defined as the energy difference between the electron conduction band minimum and the heavy-hole valence band maximum at the Γ-point of the Brillouin zone (the heavy-hole/light-hole degeneracy of the valence band is lifted owing to the strain anisotropy; see Methods). The agreement between experiment and theory is reasonably good, whereas the energy offset of 40 meV between PL and DP in Fig. 5b could be attributed to quantum confinement owing to the small diameter of the GaAs core. The heavy-hole character of the valence band was also tested with polarisation-resolved PL measurements on oriented nanowires. The polar graph in Fig. 5c shows that *E*_1_ is polarised perpendicular to the nanowire axis, as expected for recombination of electrons with heavy holes^52^.

An empirical relation that describes the change of GaAs bandgap (Δ*E*_g_) at 12 K as a function of *x* is extracted from the linear dependence of *E*_1_ on *x* in Fig. 5b:

3 $${\mathrm{\Delta }}E_{\mathrm{g}} = \left( { - 1.124 \pm 0.008} \right)\,x\,{\mathrm{eV}}$$

**Fig. 6:**
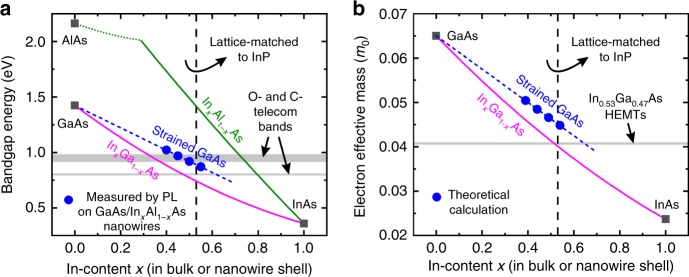
Comparison of electronic properties at the Γ-point of the Brillouin zone, at 300 K, between strained GaAs in GaAs/In_*x*_Al_1−*x*_As core/shell nanowires (our results) and strain-free III-As ternary alloys (ref. ^1^), as a function of In-content *x* in the shell or the bulk. **a** Bandgap energy. The values for strained GaAs (blue data points) are the ones for *E*_1_ measured by photoluminescence (PL) at 300 K in Fig. 5a. The blue dashed line is a linear fit. The magenta and green curves correspond to strain-free In_*x*_Ga_1−*x*_As and strain-free In_*x*_Al_1−*x*_As, respectively. **b** Electron effective mass. The values for strained GaAs (blue data points) were calculated using Eqs. (3), (4) and (5). The blue dashed line is a linear fit. The magenta curve corresponds to strain-free In_*x*_Ga_1−*x*_As. The grey bands in (**a**, **b**) indicate regimes relevant to specific device applications (telecom photonics and high electron mobility transistors-HEMTs), where typically In_*x*_Ga_1−*x*_As thin films with *x* = 0.53 (indicated with a vertical dashed line) are employed because they are lattice-matched to commercial InP substrates

We emphasise that the bandgap of GaAs at 12 K was reduced from the strain-free value of 1.52 eV to 0.91 eV for the highest strain (obtained for *x* = 0.54), i.e. a striking reduction by 40%. The same behaviour was observed at 300 K, where the bandgap energy of strained GaAs (indicated with blue arrows in Fig. 5a) was reduced to 0.87 eV with increasing *x* to 0.54. This is particularly important for applications in optical fibre telecommunications because the emission from strained GaAs nanowires can now cover the 1.3 μm (O-band) and potentially the 1.55 μm (C-band) of telecommunication wavelengths. This is better illustrated in Fig. 6a, where our results (blue data points) are also compared to the bandgap of strain-free ternary alloys^1^ (continuous curves). Although our experiments and discussion are focusing on the narrowest achievable bandgap for GaAs, all intermediate values should also be feasible by using shells with lower *L*_S_ and/or lower *x*.

A secondary PL peak (*E*_2_) was observed at 300 K (indicated with black arrows in Fig. 5a), ~40–50 meV higher in energy compared to *E*_1_. Its origin is unclear, but could be associated either with the complex radial profile of strain inside the core, which leads to complex localisation patterns of carriers^53^, or with unintentional composition/strain inhomogeneity. We also note that *E*_2_ appears even at 12 K if optical excitation power is high enough.

The reduction of the bandgap of GaAs with increasing tensile strain should be accompanied by a reduction of the effective mass of electrons at the Γ-point of the Brillouin zone. If we assume that the strain in GaAs is purely hydrostatic, the corresponding effective mass of electrons ($m_{\mathrm{e}}^ \ast$) can be estimated using the following pressure coefficients^48^:

4 $$\frac{{{\mathrm{d}}E_{\mathrm{g}}}}{{{\mathrm{d}}P}} = 12.02\,{\mathrm{eV}}/{\mathrm{Mbar}}$$

5 $$\frac{1}{{m_{\mathrm{e}}^ \ast }}\frac{{{\mathrm{d}}m_{\mathrm{e}}^ \ast }}{{{\mathrm{d}}P}} = 6.8\,{\mathrm{Mbar}}^{ - 1},$$

where d*E*_g_ is the change of the bandgap energy induced by a relative pressure d*P*. In our case, $m_{\mathrm{e}}^ \ast$ in GaAs core is expected to decrease with increasing *x* in the shell, reaching a value of $m_{\mathrm{e}}^ \ast$=0.0448 *m*_0_ at 300 K for the highest *x* here. This is equivalent to a reduction by ~30% from the strain-free value of 0.065 *m*_0_. In fact, this lowest value of $m_{\mathrm{e}}^ \ast$ is comparable to that for bulk In_*x*_Ga_1−*x*_As in the range of *x* = 0.53^1^ that is typically used in high electron mobility transistors (HEMTs) on lattice-matched InP substrates (Fig. 6b). This means that high-frequency photonics as well as high-mobility transistors could now be possible with strained GaAs nanowires and without the need for lattice-matched substrates.

## Discussion

Our results show that the GaAs core in GaAs/In_*x*_Ga_1−*x*_As or GaAs/In_*x*_Al_1−*x*_As core/shell nanowires can sustain unusually large misfit strains that would have been impossible in equivalent thin-film heterostructures. The strain of GaAs is tensile, can be engineered via the shell thickness and composition, and exhibits a predominantly hydrostatic character that is similar only to quantum-dot heterostructures. As a result, the electronic properties of GaAs can be widely tuned as if we had changed its chemical composition by adding In, but without the limitations of ternary alloys. In_*x*_Ga_1−*x*_As or In_*x*_Al_1−*x*_As shells are still necessary in order to regulate the strain in the GaAs core, but phenomena like phase separation or alloy disorder, which typically exist in ternary alloys and limit the device performance, become less critical. For the highest strain in this work, the bandgap of GaAs is reduced by 40%, making it possible to reach the 1.3 μm and potentially the 1.55 μm telecom wavelengths. A corresponding reduction of the electron effective mass by 30% is also expected. Extrapolation of our results suggests that tuning of the bandgap towards the visible range should also be possible using this time compressively strained GaAs nanowires, e.g., with GaAs_*x*_N_*1-x*_ or B_*x*_Ga_*1-x*_As shells^54,55^. Furthermore, we anticipate that the aforementioned concepts can also be adopted for other III–V binary/ternary material systems. All in all, our findings open up new possibilities for monolithically integrated III-V photonics (as well as electronics) in Si-CMOS circuits. With an advanced method of position-controlled growth, different devices (lasers, photodiodes, photovoltaic cells, etc.) could be made of the same material system on the same Si chip. The use of the same material for all devices would ensure process compatibility (e.g. no issues with cross-contamination, different processing temperatures or different thermal budget limits) and minimisation of the fabrication costs.

## Methods

### Growth of core/shell nanowires

All nanowire samples were grown by solid-source MBE. Si(111) substrates covered with a native oxide layer were subjected to an in situ surface modification process with Ga droplets to create nano-sized holes in the oxide layer for the subsequent nucleation of GaAs nanowires directly on Si^36^. The GaAs core nanowires were grown for 10 min at a substrate temperature of 615 °C using Ga and As_4_ beam fluxes equal to 6 × 10^13^ cm^−2^ s^−1^ and 2 × 10^15^ cm^−2^ s^−1^, respectively. The core growth was interrupted by closing the Ga shutter and the substrate temperature was ramped down to 370 °C under continuous exposure to the As beam. During that stage, the Ga droplets at the nanowire tips were converted to GaAs^56^. The shell growth was performed at 370 °C using In and Ga or Al beams with a total flux of 5 × 10^14^ cm^−2^ s^−1^ and an As_4_ or As_2_ beam flux of 4–5 × 10^15^ cm^−2^ s^−1^. The shell In-content *x* was controlled via the ratio of In and Ga or Al fluxes, whereas the shell thickness was controlled via the shell growth duration. The In_*x*_Al_1−*x*_As shells were capped with a 5-nm-thick In_*x*_Ga_1−*x*_As shell to avoid oxidation of the Al-containing shells in air.

### Chemical analysis

Chemical analysis based on EDXS was performed in scanning TEM mode. In particular, spectrum imaging analysis based on EDXS was used to obtain the element distributions along or perpendicular to the axis of single nanowires. Figure 1c, d, f, g show two-dimensional element distributions. Quantification of the recorded element maps including Bremsstrahlung background correction based on the physical TEM model, series fit peak deconvolution, and application of tabulated theoretical Cliff-Lorimer factors as well as absorption correction was done for the elements In (Lα line), Ga (Kα line), and As (Kα line) using the ESPRIT software version 1.9 (Bruker). The line scan plotted in Fig. 1c shows the chemical composition of the shell along the nanowire axis. The data have been extracted from the complete two-dimensional element map shown in the inset by selecting the data along a line (line width = 10 nm) parallel to the nanowire axis and in a distance of ~20 nm from the nanowire core (to exclude any contribution from the core). For the statistical confirmation of the results, we performed multiple line scans within the shell of the same or different nanowires.

### Raman scattering spectroscopy

Micro-Raman scattering measurements were performed using a frequency-doubled Nd:YAG laser with *λ* = 532 nm and 0.08 mW (the laser spot size was approximately 800 nm). Nanowires were transferred from the original substrate onto an Au-coated Si wafer. Single nanowires lying on their $\left\{ {1\bar 10} \right\}$ sidewall were selected and measured using an objective lens ×100 in back scattering and normal incidence configuration. The nanowire axis was oriented parallel to the linear polarisation of the laser, whereas the measurement was polarisation-unresolved. Different polarisation configurations were also tested and found to be in agreement with the selection rules for zinc blende nanowires^41^ (see Supplementary Fig. 2b and Supplementary Note 2). Furthermore, no signal related to wurtzite phase could be detected within the resolution of our setup (see also Supplementary Fig. 2c and Supplementary Note 2). The identification of the GaAs TO phonon peak in a Raman spectrum of a GaAs/In_*x*_Al_1−*x*_As nanowire is simple because no other peaks exist in the same range of wavenumbers (see Supplementary Fig. 4). In contrast, the close proximity of GaAs and GaAs-like TO phonons in GaAs/In_*x*_Ga_1−*x*_As nanowires (as shown in Fig. 2a) makes their distinction difficult. To overcome this problem, we used the peak position of GaAs TO phonon in GaAs/In_*x*_Al_1−*x*_As nanowires as a reference for the identification of the corresponding peak in GaAs/In_*x*_Ga_1−*x*_As nanowires with the same In-content *x* (a similar peak position is expected). This methodology was eventually validated via the successful comparison to XRD and PL results (Figs. 2c and 5b). Raman spectra from GaAs/In_*x*_Al_1−*x*_As nanowires also showed the existence of only one peak for GaAs TO phonons within the resolution of our scans. This means that the triple degeneracy of TO phonons was not clearly lifted, which is in agreement with the predominantly hydrostatic character of the strain inside the GaAs core. The peak fitting was performed with Lorentzian profiles. The error bars in Raman shift measurements (Figs. 2b and 4a) represent the standard deviation of measurements on several nanowires from the same sample (≈0.5 cm^−1^) and the instrument error (≈1.0 cm^−1^).

### Photoluminescence spectroscopy

Photoluminescence measurements were performed with a frequency-doubled Nd:YAG laser with *λ* = 532 nm and a liquid-nitrogen-cooled InGaAs detector with a response up to 1.5 μm. The excitation power was 10 mW at 12 K (to avoid heating effects) and 20 mW at 300 K for all samples in Fig. 5a (the laser spot size was ~1 mm). Nanowires were transferred from the original substrate onto a Ge wafer, which was previously implanted with Ge-ions to quench its luminescence. The measurements were performed on ensembles of a few hundreds of nanowires at various temperatures using a closed-cycle He-cryostat. Due to technical restrictions of the setup, it was not possible to perform the measurements at 12 K and 300 K at exactly the same position on the sample. Thus, the number of probed nanowires was probably different for the two temperatures. The peak fitting was performed with Voigt profiles and the full-width at half-maximum (FWHM) is shown as error bars in Fig. 5b. For polarisation-resolved measurements, a *λ*/2 plate and a polarizer were placed in the light path before and after the sample, respectively.

### High-resolution X-ray diffraction

The high-resolution X-ray diffraction characterisation was carried out at beamline P08, at the PETRA III synchrotron in Hamburg (Germany), and at beamline I07, at the Diamond Light Source in Didcot (United Kingdom). The out-of-plane symmetric reflection $\bar 1\bar 1\bar 1$ and the asymmetric reflections $\bar 3\bar 3\bar 1$, $\bar 4\bar 2\bar 2$ (cubic) and $\bar 101\bar 5$ (hexagonal) were measured in coplanar geometry at beamline P08. Here, a set of Be focusing lenses (two lenses with 0.2 mm radius), 33 m upstream from the sample, was used to define a beam size of 200 µm (vertical) × 300 µm (horizontal) at an energy of 10 keV. A two-dimensional detector Pilatus 300 K was used to collect full 3D reciprocal space maps of the signals under investigation. The in-plane $20\bar 2$ and $22\bar 4$ reflections (about 30° apart from each other) were measured in non-coplanar grazing incidence X-ray geometry at beamline I07. Here an incident angle of 0.2° was chosen close to the critical angle of total external reflection. According to the penetration depth profile of X-rays for the material under investigation, this choice ensures a depth sensitivity of only a few nanometres below the surface, and reduces significantly the diffracted contribution from the growth substrate. Also for those measurements, an X-ray beam with energy of 9 keV was similarly focused to hundreds of µm size. We optimised these parameters to be able to separate the different contributions of the core and shell in the diffraction patterns. However, the final beam size in the vertical direction corresponded to the large footprint of the X-ray beam impinging on the substrate surface, a footprint which was several mm long. Therefore, due to the given experimental conditions, the in-plane reflections show a different resolution in reciprocal space, which is mainly influenced by the illuminated sample area. Furthermore, a small thickness fluctuation of the nanowire affects much more strongly the in-plane $20\bar 2$ than the in-plane $22\bar 4$ reflections. 3D reciprocal space maps were collected using a 100 K Pilatus detector. For all samples, a constant He flux was blown around the sample within a Kapton® dome to limit possible radiation damage.

The three components of the wave vector transfer are defined as: $Q_{\left[ {\bar 1\bar 1\bar 1} \right]}$ out-of-plane component parallel to the surface normal; $Q_{\left[ {1\bar 10} \right]}$ and $Q_{\left[ {11\bar 2} \right]}$ perpendicular in-plane components. For all Bragg signals originating from the nanowires, the corresponding signal from the Si substrate was collected and it constitutes the reference of our measurements. As a last qualitative evaluation, the polytype sensitive reflections $\bar 3\bar 3\bar 1$, $\bar 4\bar 2\bar 2$ and $\bar 101\bar 5$, sensitive to zinc blende, twinned zinc blende and wurtzite, respectively, reveal twinning together with an insignificant presence of the wurtzite polytype.

2D reciprocal space maps were calculated by integrating the 3D maps along $Q_{\left[ {11\bar 2} \right]}$ for the out-of-plane $\bar 1\bar 1\bar 1$ reflection or along $Q_{\left[ {\bar 1\bar 1\bar 1} \right]}$ for the in-plane $20\bar 2$ and $22\bar 4$ ones. A further integration within a range of 0.03 1/Å perpendicular to the aforementioned directions provided 1D plots of integrated intensity vs $Q_{[\bar 1\bar 1\bar 1]}$for the out-of-plane reflection or integrated intensity vs $Q_{\left[ {10\bar 1} \right]}$ or $Q_{\left[ {11\bar 2} \right]}$ for the in-plane ones. The extracted line profiles reveal multiple diffracted contributions from nanowires and parasitic layer. In the symmetric out-of-plane reflection, nanowire core and shell appear convoluted in a common signal, while the in-plane data reveal both signals from the nanowire core and shell in a multiple peak configuration. All curves have been fitted with the help of multiple Lorentzian functions, with the intent to separate the contribution from core, shell and parasitic layer to the diffracted signal. The extracted out-of-plane and in-plane values were used to calculate the corresponding average lattice parameters shown in Fig. 3b.

We have selected the $22\bar 4$ in-plane reflection to compare calculated and experimental 1D diffraction curves. A simple kinematical approximation was used for the calculations, mainly paying attention to the effect of strain (as calculated by continuum elasticity theory) and the behaviour of the core and the shell peak positions as a function of the shell thickness. The intensity of the scattered signal can be evaluated using the following kinematical approach

6 $$I\left( {\mathbf{Q}} \right) = \left| {\mathop {\sum }\limits_{\mathrm{j}} f_{\mathrm{j}}({\mathbf{Q}})e^{ - {\mathrm{i}}{\mathbf{Q}}({\mathbf{r}}_{\mathrm{j}} - {\mathbf{u}}_{\mathrm{j}})}} \right.|^2$$

The sum is carried out over all nodes (all atoms in all unit cells of the nanowire) of the simulated strain profile, **r**_j_ is the initial unstrained positional vector of the j-th node and **u**_j_ is the corresponding displacement vector. For a nanowire of infinite length, the calculation for the in-plane $20\bar 2$ and $22\bar 4$ reflections collapses into the following one-dimensional equation:

7 $$I\left( {Q_{{\mathrm{hkl}}}} \right) = \left| {\mathop {\sum }\limits_{\mathrm{j}} f_{\mathrm{j}}(Q_{{\mathrm{hkl}}})e^{ - {\mathrm{i}}Q_{{\mathrm{hkl}}}(r_{\mathrm{j}} - u_{\mathrm{j}})}} \right.|^2\,{\mathrm{with}}\,{\mathrm{hkl}} = 20\bar 2,\,22\bar 4$$

Figure 3a shows that both the experimental data and the theoretically calculated curve exhibit the same trend. With increasing the shell thickness, the In_0.20_Ga_0.80_As peak gets more intense and shifts toward the In_0.20_Ga_0.80_As strain-free position, whereas the GaAs peak moves away from the GaAs strain-free position and becomes weaker, until it almost disappears.

### Theoretical calculations

The distribution of strain in core/shell nanowires (with infinite length) was calculated with the finite-element continuum elasticity model featured in the commercial software “nextnano”. A description of the model is given in ref. ^18^. The results were used as a reference for the analysis of the XRD data.

For the calculation of the effect of strain on the bandgap of GaAs, we used the band-edge deformation potential theory as explained in refs. ^49,51^. The effect of hydrostatic strain on the bandgap energy of GaAs was calculated with equations similar to Eq. (2). The additional change of the bandgap (Δ*E*_v_) due to strain anisotropy, which lifts the degeneracy of the valence band at the Γ-point of the Brillouin zone, was calculated according to ref. ^49^:

8 $$\Delta E_{{\mathrm{v}}({\mathrm{hh}})} = - \frac{1}{2}\delta E_{111}\quad ,{\mathrm{for}}\,{\mathrm{heavy}}\,{\mathrm{holes}}$$

9 $${\mathrm{\Delta }}E_{{\mathrm{v}}({\mathrm{lh}})} = - \frac{1}{2}{\mathrm{\Delta }}_0 + \frac{1}{4}\delta E_{111} + \frac{1}{2}\left[ {{\mathrm{\Delta }}_0^2 + {\mathrm{\Delta }}_0\delta E_{111} + \frac{9}{4}\left( {\delta E_{111}} \right)^2} \right]^{1/2}\,,{\mathrm{for}}\,{\mathrm{light}}\,{\mathrm{holes}}$$

and

10 $$\delta E_{111} = 2\sqrt 3 d\frac{{\varepsilon _{{\mathrm{zz}}} - \varepsilon _{{\mathrm{xx}}}}}{3},$$

where *Δ*_0_ = 0.34 eV is the spin-orbit splitting at the top of the valence band of bulk GaAs and *d* = −4.5 eV is the deformation potential of GaAs.

Electronic structure calculations have been performed using density-functional theory (DFT), as implemented in the ABINIT code^57^. We used the local density approximation (LDA) and Hartwigsen-Goedecker-Hutter (HGH) pseudopotentials^58^, and the results were consistent with a previous report on GaAs^59^. The results are obtained using an energy cut-off of 20 Ha and a force convergence threshold of 10^−6^ Ha/Bohr. The irreducible Brillouin zone was sampled using a set of 8 × 8 × 8 k points. The effect of spin-orbit coupling (SOC) was taken into account in the electronic structure calculations. The GW calculations are performed using self-consistent quasiparticle (QP) method on both energies and wave functions. Our QP calculations include self-consistent screened exchange approximation to the self-energy^60^. The computational details for obtaining the imaginary part of the self-energy are the same as in DFT calculations.

## Supplementary information

Supplementary Information

## Data Availability

All data are available from the corresponding author upon reasonable request.
