# Supplementary material for: Widely tunable GaAs bandgap via strain engineering in core/shell nanowires with large lattice mismatch
Source: Nat Commun. 2019 Jun 26;10:2793. doi: 10.1038/s41467-019-10654-7 (PMC6595053; doi:10.1038/s41467-019-10654-7)
Supplement: Supplementary file 1 — Supplementary Information [file 41467_2019_10654_MOESM1_ESM.pdf]

Widely tunable GaAs bandgap via strain engineering in core/shell nanowires with large lattice mismatch

− Supplementary Information −

L. Balaghi[1, 2], G. Bussone[3], R. Grifone[3], R. Hübner[1], J. Grenzer[1], M. Ghorbani-Asl[1], A. V. Krasheninnikov[1], H. Schneider[1], M. Helm[1, 2], E. Dimakis[1,⋆]

[1] Institute of Ion Beam Physics and Materials Research, Helmholtz-Zentrum Dresden-Rossendorf, 01328 Dresden, Germany

[2] Center for Advancing Electronics Dresden (cfaed), Technische Universität Dresden, 01062 Dresden, Germany

[3] PETRA III, Deutsches Elektronen-Synchrotron (DESY), 22607 Hamburg, Germany

[⋆]contact information: e.dimakis@hzdr.de

Supplementary Figures

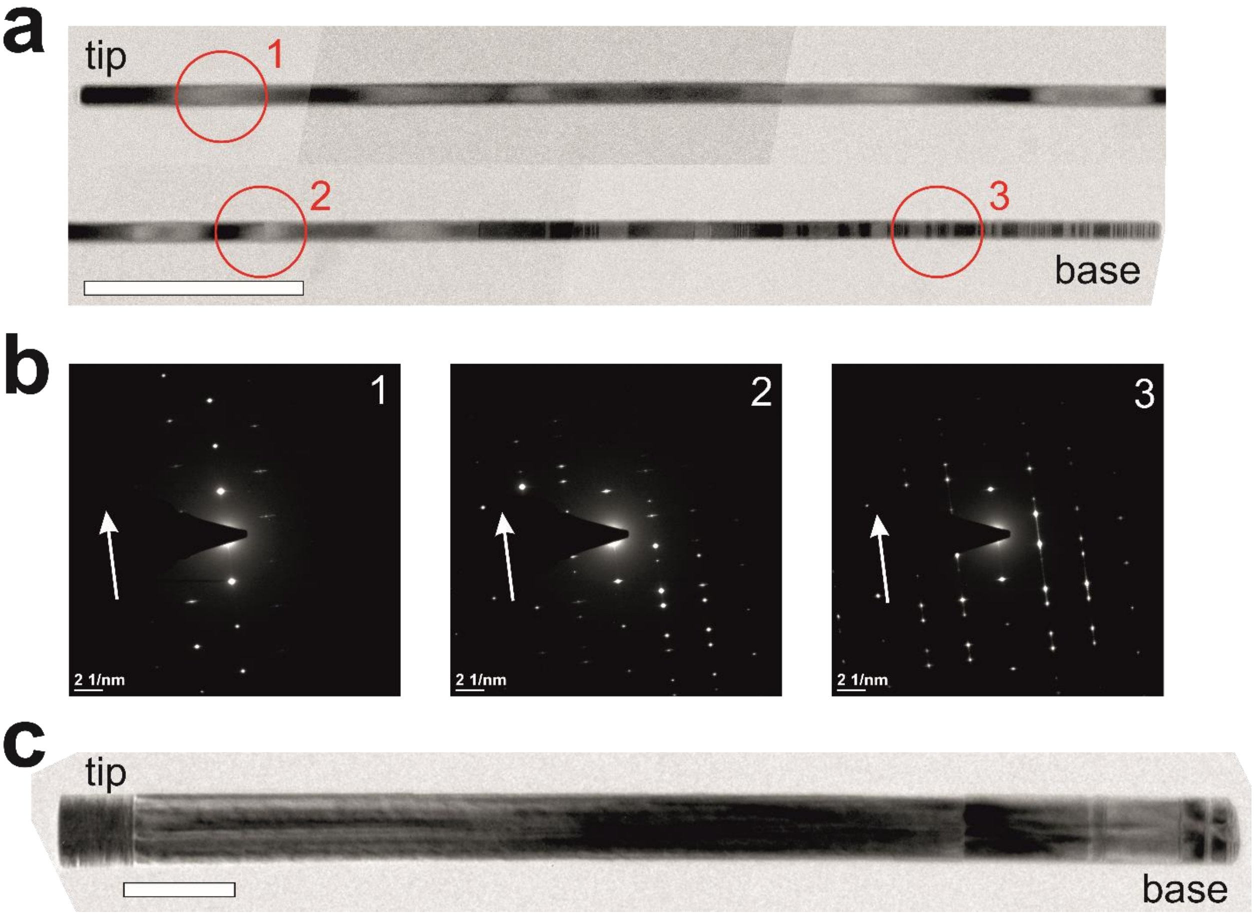

Supplementary Figure 1. Structural analysis of a core and a core/shell nanowire. (a) Transmission electron microscopy (TEM) image of a GaAs nanowire (core) without a shell. The image is split for the sake of clarity. (b) Selected-area electron diffraction patterns at the positions 1, 2, and 3 indicated with red circles in (a). The arrows indicate the growth direction. The crystal grows in zinc blende phase, whereas a high number of twins and stacking faults exist at the base of the nanowire. (c) TEM of a GaAs/$In_xGa_{1-x}As$ core/shell nanowire with shell In-content x=0.20 and shell thickness L_S=40 nm. The crystal phase of the shell follows the one of the core. In addition, a high density of twins and stacking faults exist at the nanowire tip. The scale bars in (a) and (c) correspond to 100 nm.

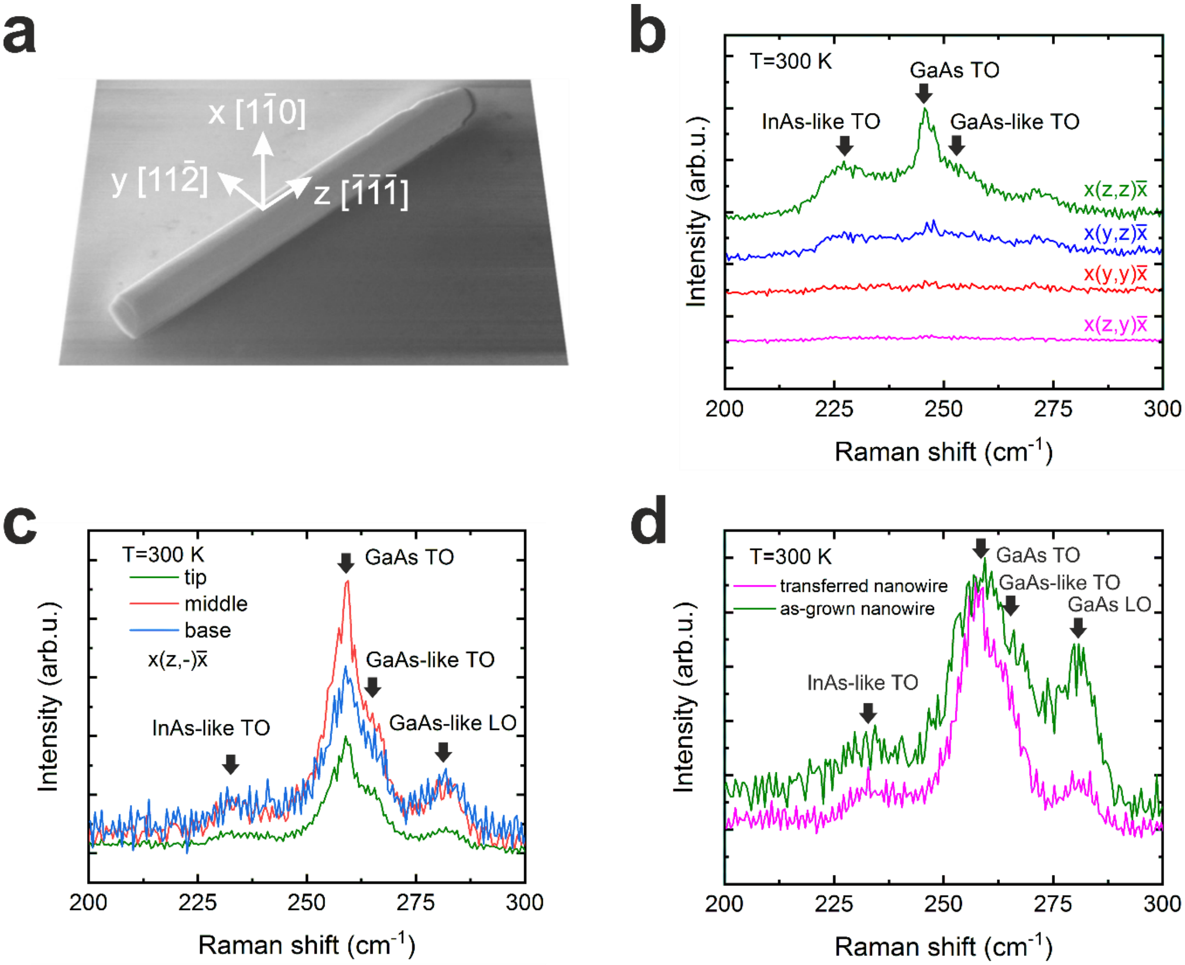

Supplementary Figure 2: Raman spectra from single nanowires at 300 K. (a) Definition of reference axes using a bird-eye view scanning electron microscopy (SEM) image of a core/shell nanowire. The nanowire has been transferred on an Au-coated Si wafer. (b) Raman spectra from a GaAs/$In_xGa_{1-x}As$ nanowire (with shell In-content x=0.45 and shell thickness L_S=40 nm) for the four main polarization configurations. The spectra have been shifted vertically for the sake of clarity. (c) Raman spectra close to the tip, the middle, and the base of the same GaAs/$In_xGa_{1-x}As$ nanowire (x=0.20 and L_S=40 nm). (d) Raman spectra from single as-grown and transferred GaAs/$In_xGa_{1-x}As$ nanowires (x=0.20 and L_S=40 nm). The arrows in all spectra indicate the various types of transverse (TO) and longitudinal (LO) optical phonons.

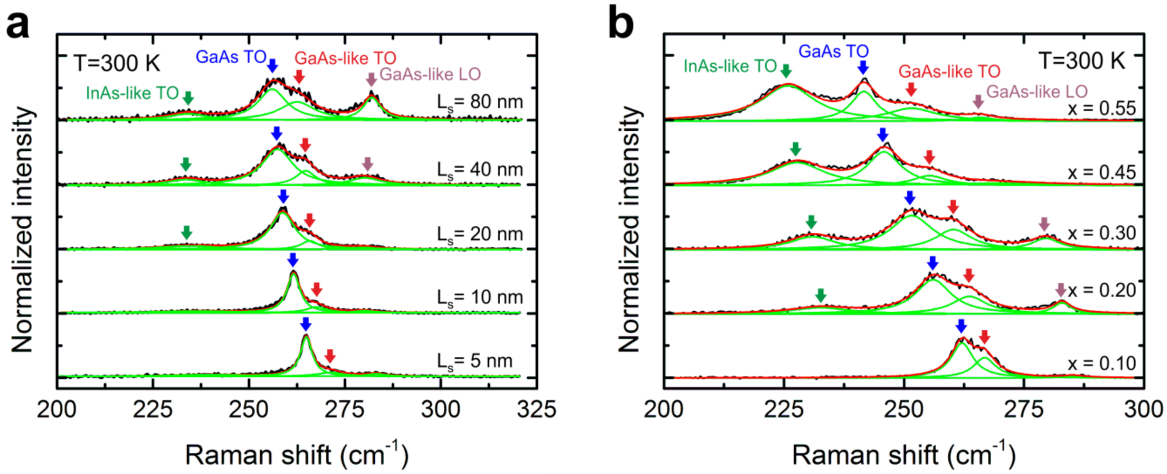

Supplementary Figure 3. Raman spectra from single GaAs/$In_xGa_{1-x}As$ nanowires at 300 K. (a) The different spectra correspond to nanowires with different shell thickness L_S (shell In-content x=0.20). (b) The different spectra correspond to nanowires with different shell In-contents x (shell thickness L_S= 40 – 80 nm). The spectra have been shifted vertically for the sake of clarity. The measured spectra (black lines) were fitted with Lorentzian profiles (green lines for the contributing peaks and red lines for the cumulative curves). The arrows in all spectra indicate the various types of transverse (TO) and longitudinal (LO) optical phonons.

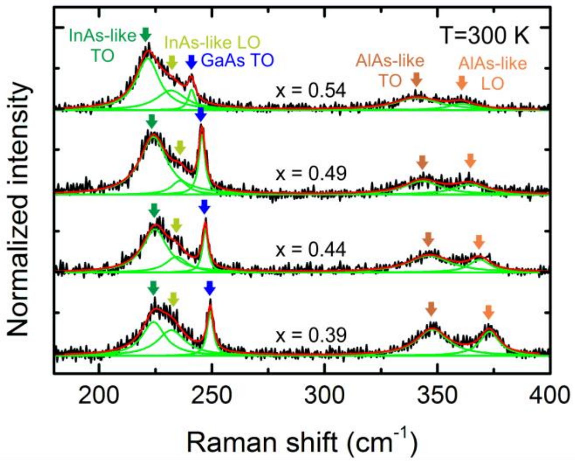

Supplementary Figure 4. Raman spectra from single GaAs/$In_xAl_{1-x}As$ nanowires at 300 K. The different spectra correspond to nanowires with different shell In-contents x (shell thickness L_S= 80 nm). The spectra have been shifted vertically for the sake of clarity. The measured spectra (black lines) were fitted with Lorentzian profiles (green lines for the contributing peaks and red lines for the cumulative curves). The arrows in all spectra indicate the various types of transverse (TO) and longitudinal (LO) optical phonons.

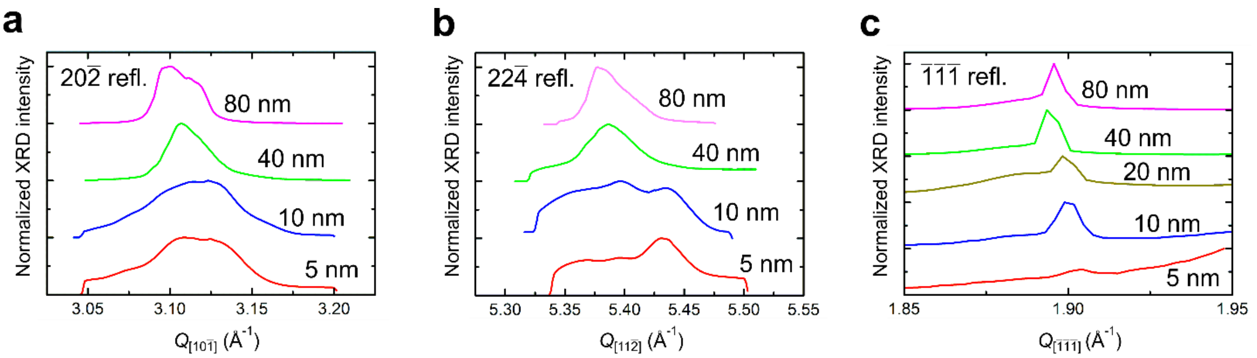

Supplementary Figure 5. X-ray diffraction spectra from GaAs/$In_{0.20}Ga_{0.80}As$ nanowire ensembles. Three-dimensional reciprocal space maps were first recorded around the reflections $20\overline{2}$, $22\overline{4}$ and $\overline{1}1\overline{1}$. Then the intensity was integrated (a) along $Q_{[\overline{1}\,\overline{1}\,\overline{1}]}$ and $Q_{[1\overline{2}1]}$ for the $20\overline{2}$ reflection, (b) along $Q_{[\overline{1}\,\overline{1}\,\overline{1}]}$ and $Q_{[1\overline{1}0]}$ for the $22\overline{4}$ reflection, and (c) along $Q_{[1\overline{1}0]}$ and $Q_{[11\overline{2}]}$ for the $\overline{1}1\overline{1}$ reflection. Spectra with different colours correspond to measurements on nanowires with different shell thickness L_S= 5 (red), 10 (blue), 40 (green) and 80 (magenta) nm. The spectra have been shifted vertically for the sake of clarity.

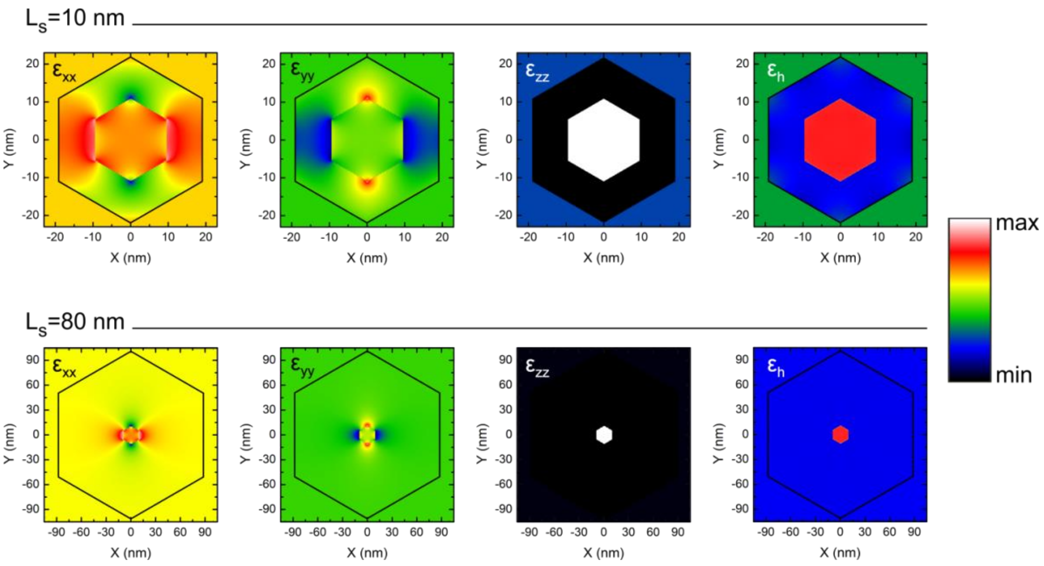

Supplementary Figure 6. Calculated distribution of strain in core/shell nanowires. Calculated strain tensors ε_{xx}, ε_{yy}, ε_{zz}, and $\varepsilon_{\mathrm{h}} = \varepsilon_{\mathrm{xx}} + \varepsilon_{\mathrm{yy}} + \varepsilon_{\mathrm{zz}}$ in a GaAs/$In_{0.20}Ga_{0.80}As$ nanowire with shell thickness L_S=10 nm (top series of plots) or L_S=80 nm (bottom series of plots), and infinite length. For L_S=10 nm, the colour scale (min, max) corresponds to: (-2.16 %, 1.25 %) for $\varepsilon_{\mathrm{xx}}$, (-1.43 %, 1.90 %) for $\varepsilon_{\mathrm{yy}}$, (-0.38 %, 1.05 %) for $\varepsilon_{\mathrm{zz}}$, and (-1.10 %, 1.96 %) for ε_{h}. For L_S=80 nm, the colour scale (min, max) is: (-1.55 %, 1.05 %) for $\varepsilon_{\mathrm{xx}}$, (-1.11 %, 1.40 %) for $\varepsilon_{\mathrm{yy}}$, (-0.02 %, 1.42 %) for $\varepsilon_{\mathrm{zz}}$, and (-0.59 %, 2.44 %) for ε_{h}. The colour for zero strain in each plot is the one shown outside of the hexagonal nanowire area. Positive values correspond to tensile strain, whereas negative ones to compressive strain. The finite-element continuum elasticity model featured in the commercial software "nextnano" was employed for the calculations.

Supplementary Notes

Supplementary Note 1. Crystal structure of core/shell nanowires

The crystal structure of the nanowires was characterized by transmission electron microscopy (TEM). The GaAs core (without a shell) grows in zinc blende phase as shown in Supplementary Figures 1a-b. The high number of rotational twins around the nanowire axis and stacking faults close to the nanowire base is attributed to a gradual increase of the Ga droplet contact angle in the beginning of the growth. Contact angles in the range of 130° favour the formation of zinc blende phase, whereas smaller angles may result in twins, stacking faults, or even wurtzite segments.

The $In_xGa_{1-x}As$ and $In_xAl_{1-x}As$ shells adopt the crystal phase of the core. A representative example of a GaAs/$In_xGa_{1-x}As$ core/shell nanowire with x=0.20 and L_S=40 nm is shown in Supplementary Figure 1c. The high number of twins and stacking faults at the nanowire tip were already formed at the end of the core growth, when the Ga droplet was exposed to the As beam and converted to GaAs. The gradual decrease of the Ga droplet contact angle caused the formation of twins and stacking faults that passed to the shell. Small wurtzite segments (i.e. continuous formation of rotational twins) could be found by high-resolution TEM only occasionally and only at the two ends of the nanowires, but their volume was negligible compared to the total volume of the nanowires.

Supplementary Note 2. Raman scattering spectroscopy on core/shell nanowires

The strain in core/shell nanowires was measured by micro-Raman scattering spectroscopy at 300 K (λ=532 nm). The measurements were performed in a back scattering configuration with normal incidence excitation $\mathrm{x}(-,-)\bar{\mathrm{x}}$ on single nanowires (the reference axes are defined in Supplementary Figure 2a), which had been transferred previously on an Au-coated Si wafer.

Supplementary Figure 2b shows Raman spectra from a GaAs/$In_xGa_{1-x}As$ nanowire (x=0.45 and L_S=40 nm) in the four main configurations, i.e. with the incident and measured light (i) polarized parallel to the nanowire axis: $\mathrm{x}(\mathrm{z},\mathrm{z})\bar{\mathrm{x}}$, (ii) polarized perpendicular to the nanowire axis: $\mathrm{x}(\mathrm{y},\mathrm{y})\bar{\mathrm{x}}$, (iii) cross-polarized: $\mathrm{x}(\mathrm{z},\mathrm{y})\bar{\mathrm{x}}$ or $\mathrm{x}(\mathrm{y},\mathrm{z})\bar{\mathrm{x}}$. In agreement with the selection rules for zinc blende GaAs in the specific geometry, the transverse optical (TO) phonon modes related to GaAs are strong for $\mathrm{x}(\mathrm{z},\mathrm{z})\bar{\mathrm{x}}$ and much weaker or absent for the other configurations. Furthermore, the E_2^H mode related to wurtzite GaAs is never observed. The Raman spectra did not show any dependence on the measurement position along the nanowire axis. This is demonstrated in Supplementary Figure 2c, where Raman spectra close to the tip, the middle, and the base of the same GaAs/$In_xGa_{1-x}As$ nanowire (x=0.20 and L_S=40 nm) are shown. Thus, the presence of twins and stacking faults at the two ends of the nanowires (as measured by TEM) did not affect the Raman spectra within the resolution of our setup. Finally, Supplementary Figure 2d shows that Raman spectra from single as-grown and transferred nanowires are very similar (small differences of 1-2 cm^{-1} in the peak positions are much smaller than the strain-induced shifts and comparable with the

instrument error and the standard deviation of measurements on many nanowires from the same sample). This means that the removal of the nanowires from the original substrate did not change their strain state. Of course, we cannot exclude a local change of the strain, below the resolution of our setup, at the nanowire base.
